# Enhanced renal clearance impacts levetiracetam concentrations in patients with traumatic brain injury with and without augmented renal clearance

**DOI:** 10.1186/s12883-023-03515-w

**Published:** 2024-01-02

**Authors:** Aaron M. Cook, Kaylee Hall, Jimmi Hatton Kolpek, Kathryn A. Morbitzer, J. Dedrick Jordan, Denise H. Rhoney

**Affiliations:** 1 Department of Pharmacy Practice and Science, University of Kentucky College of Pharmacy, Lexington, KY USA; 2 Pharmacy Services UKHealthCare, Lexington, KY USA; 3 Division of Practice Advancement and Clinical Education, UNC Eshelman School of Pharmacy, University of North Carolina at Chapel Hill, Campus Box 7574, Chapel Hill, NC 27599 USA; 4 Departments of Neurology and Neurosurgery, Duke University, Durham, NC USA

**Keywords:** Augmented renal clearance, Creatinine clearance, Traumatic brain injury, Pharmacokinetics, Levetiracetam, Seizure prophylaxis

## Abstract

**Background:**

The purpose of this study was to examine the impact of ARC on levetiracetam concentrations during the first week following acute TBI. The hypothesis was levetiracetam concentrations are significantly lower in TBI patients with augmented renal clearance (ARC) compared to those with normal renal clearance.

**Methods:**

This is a prospective cohort pharmacokinetic study of adults with moderate to severe TBI treated with levetiracetam during the first week after injury. Serial blood collections were performed daily for analysis of levetiracetam, cystatin C, and 12-hr creatinine clearance (CrCl) determinations. Patients were divided into two cohorts: with (CrCl ≥130 ml/min/1.73 m^2^) and without ARC.

**Results:**

Twenty-two patients with moderate to severe TBI were included. The population consisted primarily of young male patients with severe TBI (mean age 40 years old, 68% male, median admission GCS 4). Each received levetiracetam 1000 mg IV every 12 h for the study period. ARC was present in 77.3% of patients, with significantly lower levetiracetam concentrations in ARC patients and below the conservative therapeutic range (< 6mcg/mL) for all study days. In patients without ARC, the serum concentrations were also below the expected range on all but two study days (Days 4 and 5). Four of the 22 (18.2%) patients exhibited seizure activity during the study period (two of these patients exhibited ARC). Cystatin C concentrations were significantly lower in patients with ARC, though the mean for all patients was within the typical normal range.

**Conclusions:**

ARC has a high prevalence in patients with moderate to severe TBI. Levetiracetam concentrations after standard dosing were low in all TBI patients, but significantly lower in patients with ARC. This study highlights the need to consider personalized drug dosing in TBI patients irrespective of the presence of ARC.

**Clinical trial registration:**

This study was registered at cliicaltrials.gov (NCT02437838) Registered on 08/05/2015, https://clinicaltrials.gov/ct2/show/NCT02437838.

**Supplementary Information:**

The online version contains supplementary material available at 10.1186/s12883-023-03515-w.

## Introduction

Critically ill patients suffer high rates of medical complications, including infections (up to 51%), venous thromboembolism (up to 31%), and seizures (up to 28%) [1–3]. Treatment regimens designed to manage or prevent these complications may be compromised by altered drug concentrations because of the hyperdynamic conditions accompanying acute trauma [4, 5]. This hyperdynamic state is a result of alterations in stress hormone concentrations, vascular tone, fluid status, cardiac output, and altered blood flow to major organs that may lead to changes in organ function [6, 7]. In up to 65% of critically ill patients, creatinine clearance rates of > 130 ml/min/1.73 m^2^ have been reported [6, 7]. These rates suggest the presence of augmented renal clearance (ARC) which could lead to enhanced filtration and secretion of solutes, including drugs [8, 9]. There are few tools and no biomarkers available to clinicians to predict which patients will exhibit ARC [10]. Commonly used functional biomarker measures of renal function such as serum creatinine and cystatin C (CysC) used in standard equations to estimate creatinine clearance (CrCl) have not shown good correlation to measured creatinine clearance in critically ill patients [11–13]. However, standard practice is to use these surrogate measures to guide drug dosing [14]. There is a gap in our understanding of the impact ARC has on achieving and maintaining target therapeutic drug concentrations in critically ill patients.

Patients with severe traumatic brain injury are a subset of critically ill patients at risk of ARC with up to 85% reported to experience ARC [6, 15]. Patients with acute brain injury represent a target population where treatment guidelines recommend routine administration of agents for prophylaxis of complications including deep vein thrombosis and seizures [16, 17]. Agents commonly used for prophylaxis in these patients, such as levetiracetam, are renally eliminated, which could be affected by ARC.

The effect of ARC on standard medication dosing regimens and their effectiveness in critically ill patients has not been studied in large part because of the limited routine assay availability for many agents. Levetiracetam is an antiepileptic drug (AED) that displays linear pharmacokinetics and is nearly exclusively eliminated unchanged in the urine [18]. Levetiracetam is often employed for seizure prophylaxis during the first seven days following acute brain injury [16, 19]. The pharmacokinetic profile of this agent has largely been characterized in other patient groups under non-stress circumstances [18, 20]. Levetiracetam pharmacokinetics have been described in critically ill patients without renal dysfunction, demonstrating increased clearance and lower drug exposure [21–24]. The purpose of this study was to examine the impact of ARC on levetiracetam concentrations during the first week following acute TBI. Our hypothesis was levetiracetam concentrations will be significantly lower in TBI patients with ARC compared to what would be expected in those with normal renal clearance.

## Materials and methods

### Study design/population

This was a two-center, prospective pharmacokinetic study in patients with TBI initiated on levetiracetam for seizure prophylaxis. Adult patients (18–75 years of age) were screened for eligibility if they were admitted to either the University of Kentucky or University of North Carolina Healthcare facilities with moderate to severe documented pTBI [post-resuscitation Glasgow Coma Score (GCS) 4–12)]. Patients were included if levetiracetam was initiated for seizure prophylaxis and the anticipated length of stay in the intensive care unit was greater than 48 h. Patients were initiated on levetiracetam 1000 mg IV every 12 h with or without a loading dose. Renal dysfunction (CKD Stages 3–5 and/or SCr > 1.4 mg/dL), pregnancy, need for renal replacement therapy, history of renal transplant or nephrectomy, moribund condition were exclusion criteria. Informed consent was obtained from each patient’s approved proxy prior to inclusion in the cohort. The study received IRB approval from the respective institutions. (IRB #150,114-University of North Carolina at Chapel Hill Institutional Review Board and the Office of Human Research Ethics; IRB#15-0343-F6A-Univeristy of Kentucky Human Research/Institutional Review Board).

### Study procedures

Patient demographics (age, sex, height, weight), vital signs, cerebral perfusion pressure, intracranial pressure, ventilation status, best daily GCS, fluid balance, pertinent medications (vasopressor agents, fluids, pain/sedation/agitation agents, other antiseizure drugs, mannitol), the occurrence of seizures (either clinically overt or by EEG), and complications were recorded over the seven day period. Hospital day 1 (day of admission) was characterized as study day 1. Urine was collected over 12 h for determination of creatinine clearance daily for up to seven days when the patient had an indwelling bladder catheter. Patients were categorized as having ARC if the mean measured CrCl was > 130 ml/min/1.73 m^2^ for the study period [4]. Two levetiracetam concentrations were measured daily after the morning dose (peak and trough during the dosing interval) with a planned total of 14 levetiracetam serum concentrations per patient during the seven days of study. In every case where there was a loading dose, no sampling was done on the loading day. Sampling was only done on days where there was a regular scheduled dosing regimen. The area-under-the-curve (AUC) for levetiracetam for 0–24 h was calculated using the trapezoidal rule with the terminal AUC being calculated by last concentration/elimination rate constant [25]. Serum CysC was also evaluated daily during the study period to examine a potential relationship between higher CrCl and sub-physiologic concentrations. Augmented renal clearance in trauma intensive care (ARCTIC) scores were calculated for each patient on each day [10]. The ARCTIC score is calculated by adding up the various factors to a total score: 4 points for age < 56 years, 3 points for age 56–75 years, 3 points for serum creatinine < 0.7 mg/dl, and 2 points for male sex. The following equations were used to estimate the CrCl for each patient in order to compare to the 12-hr measured creatinine clearance: Cockcroft-Gault (CG), Cockcroft-Gault standardized by body surface area (CG-BSA), Modification of Diet in Renal Disease (MDRD), Jelliffe, Hull, Chronic Kidney Disease Epidemiology Collaboration (CKD-EPI), and David-Chandler [26–32]. The occurrence of seizures was defined as either notation of overt clinical seizures or seizure activity on clinically indicated electroencephalographic (EEG) monitoring.

### Analytical procedures

Urine volumes and creatinine concentrations were reported by clinical laboratories from each enrolling facility. The serum levetiracetam concentrations were determined by ultraperformance liquid chromatography with tandem mass detection processed by the University of Kentucky College of Pharmacy laboratories (Lexington, KY) [21]. The analytic measurement range for levetiracetam was 0.8–150 mcg/mL. Samples were stored at −70 °C over the course of the study and analyzed in batches at the conclusion of the study. No samples were excluded from analysis or reporting. For the purposes of this study, the expected serum concentration range was 6–20 mcg/mL, which aligns with prior work on seizure prophylaxis in another neurocritically ill population using a similar dosing schedule [21]. Serum cystatin C samples were analyzed using ELISA assay (R&D Systems). The concentration range for the assay was 0.102–100 ng/ml. Serum creatinine measures were completed by each institution’s clinical laboratory as part of standard medical care.

### Data analysis and statistics

Power was calculated to assess the association between ARC status (those with versus those without ARC) and sub-therapeutic serum concentrations of levetiracetam (levels below 6 mcg/mL). Because repeated serum concentrations obtained from the same patient will likely be correlated, we derived power using 1000 beta-binomial simulations. We assumed that approximately 85% of patients would experience ARC at some point over 7 days and that the proportions exhibiting ARC on each day would follow a similar distribution as observed in a previous study [5]. We further assumed that about 1/3 of enrolled patients (13 patients) would not contribute 7 days of data; as a conservative assumption, we assumed these patients would contribute exactly 4 days. Then, if sub-therapeutic serum concentrations would occur on 5% of days without ARC, enrolling 40 patients would provide at least 85% power to detect an increase of 10% in days with ARC using a 2-sided test at the 0.05 level, even if the correlation between repeated serum levels from the same patient were as high as 0.8. Missing data was addressed using casewise deletion. Alpha was set at 0.05.

Summary statistics were generated, including mean values and 95% confidence intervals, by study day. The mean and variability (SD) in peak and trough serum levetiracetam concentrations and the 12-hour measured CrCl were plotted over time. The frequency of patients with levetiracetam serum concentration outside of the therapeutic range (6–20 mcg/mL) was examined. The time course of ARC is summarized graphically across the study time period. A student’s t-test was used to compare levetiracetam AUC in patients with and without ARC (p < 0.05 considered statistically significant). Simple linear regression was used to compare 12-hour measured CrCl to seven equations estimating creatinine clearance. Data analysis was performed using Microsoft Excel 2019 (Redmond, WA).

## Results

Twenty-four patients were enrolled (UK = 9; UNC = 15) in the study (Table 1). One subject was excluded after enrollment secondary to onset of acute kidney injury, while another subject was excluded due to minimal sampling and data collection. Twenty-two subjects were included in the analysis. The population consisted primarily of young male patients with severe TBI (mean age 40 years old, 68% male, median admission GCS 4). The subgroup of patients with ARC although not statistically significant were younger in age (mean 38.3 vs. 50 years) and had lower in-hospital mortality (11.8% vs. 60%) with longer median ICU (15 vs. 8 days) and hospital (15 vs. 9 day) lengths of stay. Mean fluid balance across all study days was not significantly different between those with ARC and those without ARC [ARC vs. no ARC (Day 1: 2806.6 ± 3843.9 vs. 1957.8 ± 1745.4 mL) (Day 2: 1780 ± 1619.8 vs. 1756.5 ± 760.6 mL) (Day 3: 987.8 ± 1212.5 vs. 1619.4 ±1536.7 mL) (Day 4: 1013.2 ± 970.3 vs. 582.0 ± 1397.8) (Day 5: 586.3 ± 1173.3 vs. -698.2 ± 2051.4 mL) (Day 6: 223.2 ± 1477.6 vs. 636.6 mL) (Day 7: 186.0 ± 1664.8 vs. 2131.4 ± 2443.3 mL).

**Table 1:** Patient characteristics

Characteristic	ARC (N = 17)	No ARC (N = 5)	P-value
Age (years)	38.3 (17.6)	50 (18.9)	0.212
Gender (N, % male)	11 (64.7%)	4 (80%)	0.52
Ethnicity (N, %)			
Caucasian	11 (64.7%)	4 (80%)	
Black	2 (11.8%)	0 (0%)	
Hispanic	2 (11.8%)	0 (0%)	
Asian	1 (5.9%)	0 (0%)	
Other/unknown	1 (5.9%)	1 (20%)	
Weight (kg)	76.9 (20)	84.8 (12.8)	0.42
BMI (kg/m^2^)	25.4 (5)	28.8 (5.4)	0.211
Admission GCS	4 (3–10)	5 (3–6)	0.829
Admission Scr (mg/dl)	0.82 (0.24)	0.96 (0.25)	0.287
SOFA score	7 (2.8)	8 (2.9)	0.508
ARCTIC score	6.8 (1.4)	6.2 (1.4)	0.025
ICU length of stay (days)	15 (4–91)	8 (3–18)	0.28
Hospital length of stay (days)	15 (4-128)	9 (3–24)	0.28
In-hospital mortality	2 (11.8%)	3 (60%)	0.055
Measured creatinine clearance (ml/min/1.73 m^2^)	190.5 (59)	122 (36)	< 0.001

Data is represented as mean (standard deviation) or median (range) unless otherwise specified

*BMI* body mass index, *GCS* Glasgow Coma score, *Scr* serum creatinine, *SOFA* sequential organ failure assessment, *ARTIC* augmented renal clearance in trauma intensive care, *ICU* intensive care unit

**Fig. 1:**
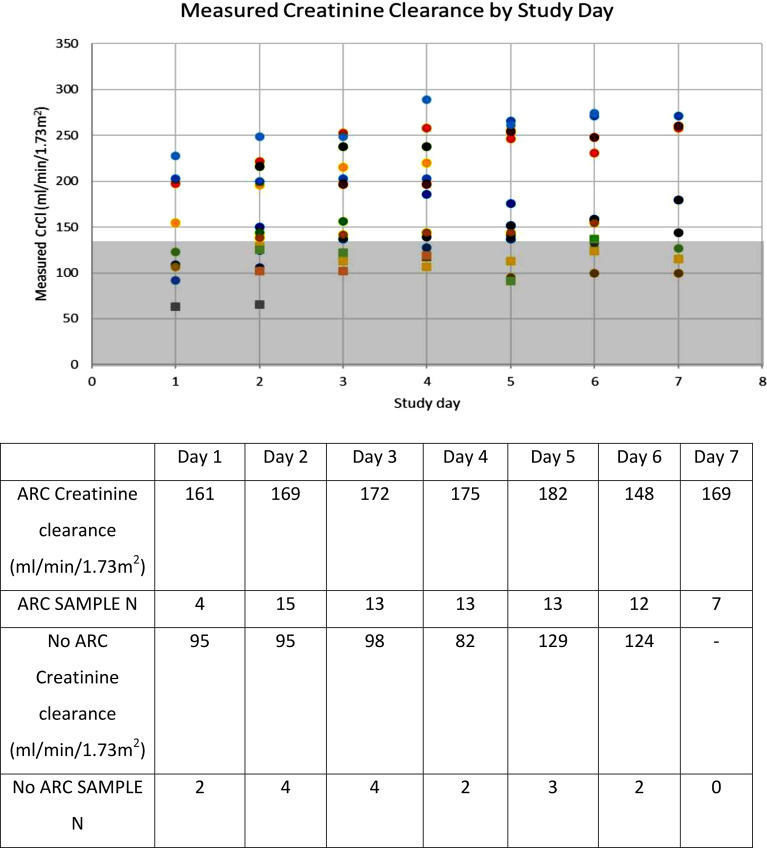
Measured creatinine clearance by study day*. Mean measured creatinine clearance by study day in patients with and without ARC. The shaded region indicates the creatinine clearance which would *not* be considered ARC (< 130 ml/min/1.73 m^2^). Overall difference between mean measured creatinine clearance was P < 0.001. *Circle data points = patients with ARC; Square data points = patients with no ARC

The mean 12-hour measured CrCl for all patients included was 157.4 ml/min/m^2^ (SD 49). Overall, 17 patients were in the ARC cohort for the study period compared to 5 patients in the no ARC cohort (ARC incidence 77.3%). The mean 12-hour measured CrCl in ARC patients was 190.5 (SD 59) ml/min/1.73 m^2^ compared to 122 (SD 36) ml/min/1.73 m^2^ in no ARC patients (p < 0.001). The mean CrCl on each day was consistently above the ARC threshold in patients with ARC and below the ARC threshold in those without ARC (Fig. 1). Only one patient in the no ARC cohort had any days with a 12-hour measured CrCl > 130 ml/min/1.73 m^2^. The mean peak CrCl occurred on hospital day 5. The median ARCTIC score for the study period was 6.8 for ARC patients and 6.2 for no ARC patients (p = 0.02).

**Fig. 2:**
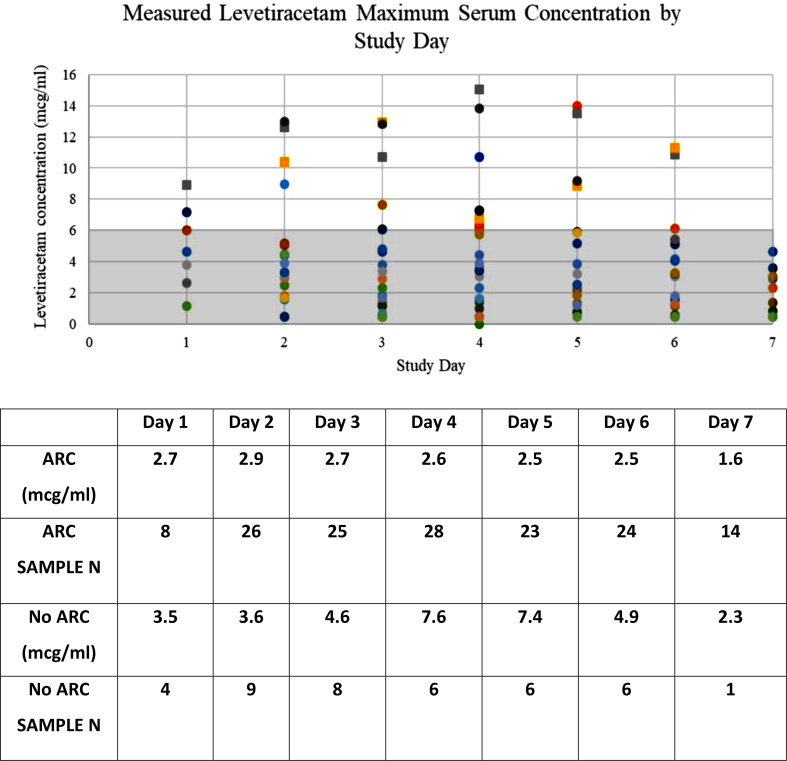
Measured levetiracetam maximum serum concentration by study day*. Summary of mean measured levetiracetam serum concentrations by study day in patients with and without ARC. *Circle data points = patients with ARC; Square data points = patients with no ARC; shaded area indicates below the typical therapeutic range

Mean levetiracetam serum concentrations were below the expected range for all study days in patients with ARC (Fig. 2). In patients without ARC, each of the serum concentrations were also below the expected range on all but two study days which were days 4 and 5. The calculated mean levetiracetam AUC was 62 (SD 40.6) ug*hr/ml in ARC patients, compared to 120.7 (SD 69) ug*hr/ml in no ARC patients (p = 0.028), confirming patients with ARC had lower overall levetiracetam exposure during the study period compared to those subjects who did not exhibit ARC. Eight of the patients (36.4%) received loading doses of levetiracetam and 100% of patient receiving the loading dose experienced ARC. The mean maximum levetiracetam concentrations on day 1 of maintenance dosing in those receiving a loading dose was 3.2 (SD 2.4) compared to 4.7 (SD 2.9) in those not receiving the loading dose (9 of the 14 patients experienced ARC). All serum samples obtained for levetiracetam assay were used and none were excluded.

Prospective EEG monitoring was not used in this study, however 4 out of the 22 patients (18.2%) exhibited clinical seizure activity that warranted an EEG. Two of the patients who exhibited seizure activity did not exhibit ARC. Median trough concentrations for these four patients were 0.7 (0–13.97) mcg/mL. One of these patients had a clinically suspected seizure that was not verified after subsequent EEG monitoring. The other patient had periodic epileptiform discharges on EEG and then a focal onset seizure the following day (on neither day did the patient exhibit ARC). The other two patients who had seizure activity were included in the ARC cohort and both patients exhibited ARC on the date of seizure activity (one patient had a suspected clinical seizure and the other had periodic epileptiform discharges on EEG).

**Fig. 3:**
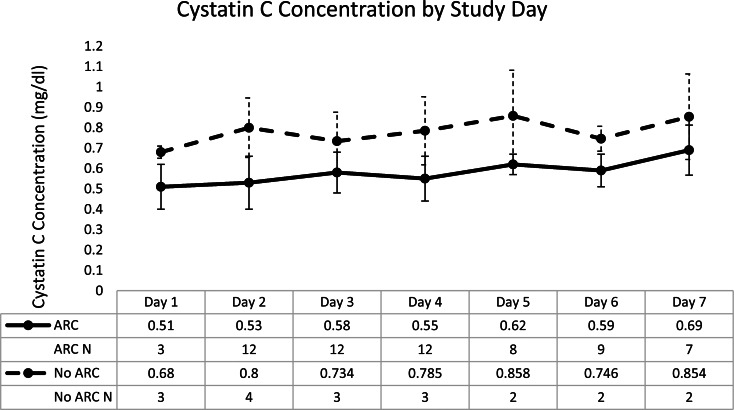
Mean cystatin C serum concentrations by study day in patients with and without ARC. The shaded region indicates the normal range for cystatin C concentration (0.53–0.92 mg/dl)

The mean Cystatin C concentrations were significantly lower in ARC patients compared to those without ARC (0.58 mg/dl vs. 0.78 mg/dl, p < 0.001, Fig. 3). However, both values were in the commonly accepted normal range (0.53–0.92 mg/dl). Common equations used to estimate CrCl all showed a poor relationship with 12-hour measured CrCl in this population (R^2^ range = 0.1642–0.394) (see Supplemental Figures). The CKD-EPI equation appeared to perform the best of all the equations, but the relationship with 12-hour measured CrCl was poor (R^2^ = 0.394).

## Discussion

Levetiracetam concentrations and overall exposure was significantly reduced in the presence of ARC in patients with TBI during our seven-day study period. The incidence of ARC in the study population was 77.3% and the mean 12-hour measured CrCl in these patients was 190.5 ml/min/1.73 m^2^. ARC persisted in this cohort throughout the seven-day study period. Levetiracetam serum concentrations were consistently below the traditionally expected range based on the dose administered and population pharmacokinetics [21, 33]. Overall, our data suggests that patients with moderate to severe TBI have lower than desired levetiracetam serum concentrations irrespective of the presence of ARC; this observation is significantly compounded by the presence of ARC, resulting in clinically relevant reduced levetiracetam exposure.

The results of the current study corroborate previously published work in this area and builds on the link between ARC and levetiracetam exposure. Spencer, et al. [21] evaluated the pharmacokinetics of levetiracetam in a heterogeneous population of neurocritical care patients [21]. In this study, only two of the twelve patients suffered TBI (the majority of patients were admitted with aneurysmal subarachnoid hemorrhage). Patients received a lower mean intravenous dose than in the current study (approximately 500 mg every 12 h). Predictably, serum levetiracetam concentrations were lower than desired, although the mean concentrations were within the expected range for much of the dosing interval. The investigators also demonstrated a modest relationship between estimated CrCl and levetiracetam clearance but did not have measured CrCl to specifically evaluate the presence of ARC. The levetiracetam concentrations in our study were consistently lower than that of Spencer, et al. despite the higher dose, both when evaluating serum concentrations and AUC. The levetiracetam serum concentrations were consistently lower in the current study regardless of the time obtained in the dosing interval compared to the trough concentration of 3.1 mcg/ml found in Spencer, et al. The AUC was similarly reduced in our study compared to Spencer, et al. despite the higher levetiracetam dosing in our study (60 mcg*hr/ml in our patients with ARC vs. 97 mcg*hr/ml). The patients included in this Spencer study would be at high risk of ARC given the acuity and diagnosis, but identification of ARC was not a component of this study. Thus, it is not clear why the levetiracetam exposure was higher in this study, though population differences in the previous study (slightly older patients, lower estimated CrCl at baseline in the Spencer cohort) may have accounted for this variance [21].

Recently Sime et al. developed a population pharmacokinetic model for levetiracetam in a mixed population of patients with severe TBI and aneurysmal subarachnoid hemorrhage [22]. From the simulated model the median trough concentration of level reduced by 50% for every 40 mL/min/1.73 m^2^ increase in measured creatinine clearance. The investigators suggested that at least 6 g/day of levetiracetam would be required for patients with ARC although the probability of trough concentration target is very low even at these increased doses [22]. In our study we used a previously reported dosing schedule for seizure prophylaxis where 2 g/day was administered [21] to patients with moderate to severe TBI. We had a large number of concentrations that were undetectable so we were unable to conduct modeling of our data similar to Sime et al. [22] to calculate clearance and provide recommendations for future dosing. When evaluating the simulations from Sime et al. [22] at measured CrCl between 161 and 181 mL/min/1.73 m^2^ which is what we reported in our ARC population, the predicted trough levetiracetam concertation was 1.1–2.6 mcg/mL which is similar to the trough concentrations we measured of 1.6–2.9 mcg/mL (in those with detectable concentrations). However, when comparing our no ARC population to the simulations reported in Sime et al. our measured CrCl on days 1–3 of 82–98 mL/min/1.73 m^2^ resulted in measured trough concentrations of 3.5–4.6 mcg/mL while the simulation predicted a trough concentration of 10 mcg/mL [22, 23]. This suggests an important new consideration of individualized dosing regimens using serum concertation and measured CrCl to guide therapy in *all* patients in the ICU with or without ARC since levetiracetam clearance is heavily related to CrCl [22, 23].

Several studies have described the high incidence of ARC in patients with acute neurologic injury. The published range of ARC incidence across a broad neurocritical population is 40 to 100%, including an incidence of 85% in TBI patients [12, 15, 34]. The variation in this incidence maybe related to varying definitions of ARC that have been defined. We used the most used definition of ARC however it is important to highlight that even in our TBI patients without ARC with a median age of 50 years the average measured CrCl was 122 mL/min/1.73 m^2^ which is higher than we may expect for patients at this age. Morbitzer et al. [12] reported enhanced renal clearance in patients with intracerebral hemorrhage and specifically highlighted a similar observation with increased measured CrCl beyond what would be expected with the age of the patients. Given that we found lower than expected levetiracetam concentrations in all patients enrolled in this study it raises the question of looking beyond the standard definition of ARC and looking towards a definition that would account for the potential enhanced renal clearance above what is expected based upon age since this enhanced renal clearance could and did impact drug concentrations in our study.

The current study demonstrates a comparable rate of ARC in moderate to severe TBI patients (77.3%), while also evaluating the effect of ARC on drug clearance across a seven day study period. Notable in this and other studies are important concepts related to drug dosing. First is the interesting, elevated CrCl resulting in hyper-elimination of medications and pharmacokinetic alterations. The markedly reduced serum levetiracetam concentrations in our cohort are similar to what has been described in previously published studies showing reduced exposure to beta-lactam antibiotics and vancomycin in patients with ARC [35–37]. The current study highlights a similar suboptimal exposure for patients with ARC to standard doses of levetiracetam. The presence of ARC merits consideration as to whether dosing medications beyond the typical ‘maximum recommended dose’ is necessary. Second are the clinical implications of ARC for patients with TBI. Lower serum concentrations of levetiracetam would be expected to lead to increased treatment failure. Serum concentrations < 6 mcg/ml may be associated with reduced efficacy in preventing seizures [38]. We use the conservative therapeutic range for levetiracetam as there are studies that also report the therapeutic range as 12–46 mcg/mL [39] in which case an even lower number of patients without ARC would not have achieved. The cohort of TBI patients in this study is too small to definitively evaluate the impact of ARC on treatment failure. However, 4 out of the 22 patients (18.2%) included did exhibit seizure activity with very low trough levetiracetam concentrations. This rate is comparable to the typical seizure rate of patients after TBI who do not receive prophylaxis, which ranges from 3.7 to 14.2% [40, 41]. The higher seizure rate in our study in patients receiving seizure prophylaxis may be influenced by low levetiracetam exposure, though the small sample size and severity of injury may also play a role. In all but one case, patients who had seizure activity had a serum levetiracetam concentration < 6 mcg/ml (in one instance in the patient who had a focal seizure, the concentration was 6.15 mcg/ml). Second, levetiracetam may be neuroprotective after TBI [42]. Low levetiracetam exposure due to ARC may negate these potential benefits without specific attention to dosing and drug exposure. Further definition of the role of levetiracetam in neuroprotection after TBI, and the necessary serum concentrations for such an effect, is needed.

One unique aspect of this study was the evaluation of a candidate biomarker to define the relationship between cystatin C, ARC, and levetiracetam concentrations. Classically, cystatin C has proved to be useful in the setting of chronic kidney disease. A biomarker to specifically identify patients with ARC would be clinically useful. Many scoring systems used to calculate the risk of ARC may not be sensitive enough and collection of urine for CrCl measurement is not timely and can be fraught with potential for error [10]. Although the cystatin C concentrations were significantly lower in patients with ARC, both cohorts exhibited concentrations within the normal range. Further evaluation of cystatin C as a biomarker for ARC is needed. Additionally, we evaluated the correlation of various common equations used in daily clinical practice to measured CrCl and found very poor relationships, which presents challenges in identifying patients with ARC without urine collection for measured CrCl. This finding is similar to what has been previously reported in the literature [14].

There are limitations to this study which merit discussion. First, the initial goal sample size was 40 patients to assess the relationship between creatinine clearance and the occurrence of subtherapeutic serum levetiracetam concentrations. Since we included only TBI the enrollment numbers were slower than we expected the decision was made to curtail subject recruitment after 24 patients. Thus, the study was underpowered although provides important data on levetiracetam concentrations in patients with TBI. Second, due to the severity of illness of our study population and their complexity of their care, some patients expired during the study period or were not appropriate or available for sampling, thus some serum collections were unavailable. In cases where data points were missing, no data was interpolated; thus some data points were evaluated for less than the total sample size (n = 22). Third we were unable to conduct the pharmacokinetic modeling due to large number of undetectable concentrations. However, based upon our data it is likely that any doses/dosing intervals calculated from this modeling would not be feasible clinically given we do not have a good method to identify when this enhanced clearance abates such that the increased dosing required could put the patient at risk for adverse effects, especially psychiatric adverse effects that have been reported in this patient population. This would suggest that LEV may not be the best drug to use in this population for seizure prophylaxis since even the Sime et al. [22] suggested a low probability of achieving goal concentrations with increased dosing. Finally, we did not use continuous EEG monitoring, thus we may not have detected subclinical seizure activity that could have occurred because of the low concentrations we reported in the enrolled patients.

In summary all 22 moderate to severe TBI patients included in this study exhibited LEV concentrations below what was expected with a large number of these patients exhibiting literature defined ARC. Our results may call into question defining ARC by a CrCl value and instead understanding enhanced renal clearance to also include values above what is expected that also impact drug concentrations which might call for a need to consider a dosage adjustment. While we do not know the biological underpinnings to enhanced clearance outside what has been described for ARC, it would be hypothesized it would be similar. Since all these TBI patients exhibited enhanced clearance and there is currently no identified biomarker the use of measured CrCl to guide LEV therapy in *all* patients in the ICU with or without the current definition of ARC would be the most ideal approach to identify those with this altered renal clearance. While we were unable to recommend LEV doses based upon our data, we did show a large number of undetectable concentrations and the potential need for increased doses above what would be considered clinically feasible, which may suggest clinicians to call into question if LEV is the most ideal agent for seizure prophylaxis.

## Conclusions

This study included 22 patients with moderate to severe TBI, the majority of whom exhibited ARC throughout the seven-day study period. Despite standard intravenous dosing of levetiracetam, serum concentrations were below the expected range in nearly every patient. Furthermore, serum levetiracetam concentrations were significantly lower in patients with ARC compared to those without. This study may have implications on levetiracetam dosing in TBI patients with and without ARC and would suggest a new to consider individualized dosing regiments using serum concentration and measured CrCl to guide therapy in all patient ins the ICU. Consideration for dose adjustment or therapeutic drug monitoring may be necessary in these patients. Future work should consider further evaluation of the concept of enhanced renal clearance outside the standard ARC definition as this study suggests there may be implications on serum drug concentrations.

### Electronic supplementary material

Below is the link to the electronic supplementary material.

**Supplementary Material 1: Supplemental Figure Panels:** Mean measured creatinine clearance compared to standard equations for estimating creatinine clearance: A. Cockcroft-Gault (CG); B. Cockcroft-Gault standardized by body surface area (CG-BSA); C. Modification of Diet in Renal Disease (MDRD); D. Jelliffe; E. Hull; F. Chronic Kidney Disease Epidemiology Collaboration (CKD-EPI); G. Davis-Chandler

## Data Availability

The datasets used and/or analyzed during the current study are available from the corresponding author on reasonable request.
