# Supplementary material for: Enhanced renal clearance impacts levetiracetam concentrations in patients with traumatic brain injury with and without augmented renal clearance
Source: BMC Neurol. 2024 Jan 2;24:12. doi: 10.1186/s12883-023-03515-w (PMC10759330; doi:10.1186/s12883-023-03515-w)
Supplement: Supplementary file 1 — Supplementary Material 1: Supplemental Figure Panels: Mean measured creatinine clearance compared to standard equations for estimating creatinine clearance: A. Cockcroft-Gault (CG); B. Cockcroft-Gault standardized by body surface area (CG-BSA); C. Modification of Diet in Renal Disease (MDRD); D. Jelliffe; E. Hull; F. Chronic Kidney Disease Epidemiology Collaboration (CKD-EPI); G. Davis-Chandler [file 12883_2023_3515_MOESM1_ESM.docx]

A Comparison of Cockcroft-Gault (CG) equation to measured creatinine clearance (CrCl)

B Comparison of Cockcroft-Gault standardized by body surface area (CG-BSA) equation to measured creatinine clearance (CrCl)

C. Comparison of Modification of Diet in Renal Disease (MDRD) equation to measured creatinine clearance (CrCl)

D. Comparison of Jelliffe equation to measured creatinine clearance (CrCl)

E. Comparison of Hull equation to measured creatinine clearance (CrCl)

F. Comparison of Chronic Kidney Disease Epidemiology Collaboration (CKD-EPI) equation to measured creatinine clearance (CrCl)

G. Comparison of Davis-Chandler equation to measured creatinine clearance (CrCl)
